# Impact of a structured HIV testing program in a hospital emergency department and a primary care center

**DOI:** 10.1371/journal.pone.0220375

**Published:** 2019-08-01

**Authors:** Cristina Gómez-Ayerbe, Javier Martínez-Sanz, Alfonso Muriel, Pilar Pérez Elías, Ana Moreno, Rafael Barea, Lidia Polo, Agustina Cano, Almudena Uranga, Cristina Santos, José Luis Casado, Carmen Quereda, Gema Robledillo, Alberto Díaz-de Santiago, María Jesús Vivancos, Fernando Dronda, Enrique Navas, Santiago Moreno, María Jesús Pérez Elías

**Affiliations:** 1 Department of Infectious Diseases, Hospital Universitario Ramón y Cajal, IRYCIS, Madrid, Spain; 2 Biostatistics Unit, Hospital Universitario Ramón y Cajal, IRYCIS, Madrid, Spain, CIBERESP, Departamento de Enfermería, Universidad de Alcalá, Madrid, Spain; 3 Centro de Salud García Noblejas, Madrid, Spain; 4 Centro de Salud Canal de Panamá, Madrid, Spain; 5 Hospital General Universitario Gregorio Marañón, Madrid, Spain; University of Ghana College of Health Sciences, GHANA

## Abstract

**Introduction:**

HIV testing guidelines are poorly implemented in most clinical settings. The best screening strategy and healthcare scenario are still unknown. The aim of our study is to evaluate the impact of a structured HIV testing intervention (DRIVE), compared to HIV testing as routinely performed in clinical practice, in two different clinical settings: a primary care center and an emergency department.

**Methods:**

Prospective evaluation of an HIV testing strategy in two clinical settings from the same healthcare area. The DRIVE program included trained nurse practitioners to perform the screening, a questionnaire to assess the risk of exposure and HIV indicator conditions (RE&IC), and rapid HIV tests. The main variables between the DRIVE program and clinical practice were the absolute number of newly diagnosed HIV infections and testing coverage.

**Results:**

The DRIVE program included 5,329 participants, of which 51.2% reported at least one positive answer in the questionnaire. The estimated HIV testing coverage was significantly higher in the DRIVE program than in the routine clinical practice (7.17% vs. 0.96%, p < 0.001), and was better in the primary care center than in the emergency department with the two strategies. Twenty-two HIV-positive people were identified, with a rate of 8.6‰ in the emergency department vs. 2.2‰ in the primary care center (p = 0.001). A higher rate of new HIV diagnoses was found in the DRIVE program compared to routine clinical practice (29.6 vs. 3.1 per 100,000 patients attended; p < 0.001).

**Conclusions:**

An easy-to-implement, structured intervention increased the absolute number of new HIV diagnoses and HIV tests, compared to routine clinical practice.

## Introduction

After more than 30 years of the HIV pandemic and great advances in HIV knowledge and antiretroviral treatment, the HIV epidemic continues to increase all over the world, causing a high number of deaths, especially in developing countries [1]. In Spain, the number of people living with HIV rises every year, as it is estimated that up to 18% of infected people are unaware of their own status [2]. These numbers are similar in other European countries [3]. In Western Europe, up to 45–50% of new HIV diagnoses are made late (with a CD4 count < 350 cells/mcl) and nearly 30% already having advanced disease at the diagnosis (< 200 CD4 cell/mcl) [4,5]. It is necessary to reduce the prevalence of people living with HIV who are unaware of their status, in order to bring down the incidence of new HIV infections [6,7].

The data above show that the current diagnostic strategies are not good enough. Unfortunately, the proven tools for HIV prevention are few [8] and difficult to implement, so we must focus on early diagnosis and early antiretroviral therapy (ART). An early HIV diagnosis implies not only personal benefits but also includes the community, since late diagnosis favors HIV transmission [9] and increases the use of resources and healthcare costs [10,11]. Maximum coverage of a screening program is essential to assure HIV “universal testing and universal treatment.” Some studies show that HIV-infected individuals who were diagnosed late presented to a healthcare facility multiple times in the two years prior to diagnosis, demonstrating a high percentage of missed diagnostic opportunities [12,13].

HIV testing guidelines stipulate different approaches, which vary from universal screening for people from 13 to 64 in all healthcare settings, unless participants decline (‘opt-out’ screening), to a targeted strategy in which HIV testing is recommended for individuals presenting with one of the indicator conditions [14–16]. Universal ‘opt-out’ testing strategies have been proposed to reduce the incidence of late diagnosis [17]. Although this non-targeted HIV testing strategy was shown to be cost-effective in populations with an HIV prevalence of at least 0.1% [18], the overall budget may be unaffordable for some countries. Furthermore, some studies exploring the routine ‘opt-out’ HIV screening strategy in Europe observed a low percentage of hidden HIV infection, as it cannot always identify early HIV infections [19]. In the United States, striking efforts have been made to establish non-targeted HIV screening in clinical practice. These operational programs have only been successful in some institutions with dedicated resources [20]; however, poor efficiency is expected in low prevalence areas [21].

The main area of discrepancy in HIV screening guidelines is the cost-effectiveness of routine testing among a population that is not known to be at higher risk [14,15]. Spanish guidelines recommend HIV testing in a variety of situations, with most implying previous risk of HIV exposure or indicator conditions, mainly but not exclusively in primary care centers [22]. Many studies have described results of HIV testing strategies in different healthcare scenarios, but few have face-to-face contact for comparison [23–25]. HIV testing in hospital emergency departments has been extensively evaluated [21,23], while sparse information is available to primary care centers or sexually-transmitted disease clinics [24,25]. Primary care centers are the health resource most frequently used by Spanish population to carry out HIV screening [26]. It is estimated that 85% of the population attend primary care every year. In addition, in this study screening has been implemented in the emergency department, since it is a good setting to perform screening in a large number of the population, especially in high risk individuals who are less likely to have consistent access to primary care, such as undocumented immigrants, drug users, and people with unstable housing [27].

The aim of our study is to evaluate the impact of a structured HIV-testing intervention (DRIVE program) in two clinical settings: a hospital emergency department and a primary care center in the same healthcare area, compared to HIV testing performed in routine clinical practice.

## Methods

### Design and study procedures

The DRIVE study (*Diagnóstico Rápido de la Infección por VIH en España*, or in English ‘Rapid HIV Testing in Spain’) is a prospective intervention program designed to investigate different aspects of HIV testing. The study was conducted in the Eastern healthcare area of Madrid (Spain), with an estimated overall population of 555,665. Primary care is distributed between 20 primary care centers, with all of them referred to the same tertiary center (Ramón y Cajal Hospital) for microbiology laboratory procedures or specialized HIV/AIDS management. People from 18 to 60 years who visited the emergency department at Ramon y Cajal Hospital or one of the primary care centers (‘Hermanos García Noblejas Primary Center’) between July 2012 and June 2013 were offered to be included in the DRIVE study. Exclusion criteria were: prior HIV diagnosis, having already been included in the DRIVE study, or inability to understand Spanish. The study was approved by the research Ethics Committee at the Ramón y Cajal Hospital and the Madrid Regional Primary Care Research Committee; all participants gave written informed consent.

A commercial rapid HIV test (INSTI, BioLytical Laboratories, Richmond, BC, Canada) with high sensitivity and specificity (> 99% for both) was performed for all participants. In addition, all participants were asked to fill out a validated HIV risk of exposure and indicator conditions (RE&IC) questionnaire at the time of inclusion in the study [28]. The questionnaire consists of 6 items that investigate the risk of exposure to HIV (based on identified HIV routes of transmission), with 14 items related to HIV indicator diseases (which are based on a selection of the ‘*HIV Indicator Diseases across Europe Study’* (HIDES) [29,30]).

In addition, routine clinical practice in the emergency department and primary care center was retrospectively investigated, using the centralized database of the Ramón y Cajal Hospital Microbiology Department laboratory to review all requested HIV enzyme immunoassays (EIA) and their results. Three periods were analyzed within routine clinical practice: the year prior to the implementation of the DRIVE program (July 2011 to June 2012), the study period (July 2012 to June 2013), and the year following DRIVE implementation (July 2013 to June 2014).

### Description of the DRIVE program

All staff in the two study settings, including medical assistants and nurses, received information about the study through written summaries, flyers, and slide presentations, which explained the main concepts of HIV screening and the study design. Participants were included through recruitment in medical or nursing consultations or through active recruitment by the study’s nurse practitioners, who were established in a specific room. Posters and brochures were placed in both locations to announce the study. In a confidential setting, after providing informed consent, all participants responded to the RE&IC questionnaire [28] and were tested for HIV with rapid tests. The study activities were carried out by the trained nurse practitioners: nine nurses in the emergency department and two nurses in the primary care center. They explained all the procedures to the participants, made sure they filled out the RE&IC questionnaire, and helped as needed. Subsequently, they numbered, performed, and photographed the rapid HIV test results. All data were transmitted to a central web database via electronic tablets. There were two project coordinators who periodically checked the database, confirmed the results with photographs, verified HIV-positive results with a Western Blot test, and discarded the existence of a previous diagnosis. Furthermore, they assessed possible errors as well as incomplete records.

Information about the number of people visiting emergency department services or primary care centers was provided by the Information Department of the Ramon y Cajal Hospital and the Healthcare Department of the Community of Madrid, respectively.

All people with an HIV-positive test in the DRIVE program were informed and counseled about their results. At that time, the nurse practitioners informed the project coordinators in the Infectious Diseases Department of the Ramon y Cajal Hospital, where they were referred for a complete HIV evaluation during the next 48 hours. An HIV EIA and a Western Blot were initially obtained. Only confirmed HIV-positive people, without evidence of a prior HIV diagnosis, were considered as new HIV diagnoses in the analyses. A first baseline CD4 cell count was considered for all patients. Diagnoses outside the DRIVE program were managed according to routine clinical practice.

### Definitions, data, and statistical analysis

Variables considered in this study were: gender, age categorized in three strata (< 30, 30–50, and > 50 years), country of birth categorized in two (Spanish or non-Spanish), setting of inclusion in the DRIVE study (emergency department or primary care center), estimated number of people between 18 to 60 years attending each facility, the rapid HIV test and the RE&IC questionnaire results (positive or negative), as well as the total number of people with an HIV EIA request to the Microbiology laboratory. A questionnaire was considered ‘positive’ if any item was answered with ‘yes,’ therefore indicating some risk for HIV infection.

Coverage was defined as the proportion of people who were tested for HIV with either rapid tests or EIA among the total number who attended. We compared coverage in both settings (the emergency department and a primary care center) and scenarios (DRIVE study and clinical practice) during the three periods studied. Due to the smaller sample size and few events occurring in the routine clinical practice arm, three years of study were grouped into a single period. As the estimated number of people attending was very similar throughout the years studied, a stable population was considered. The new HIV diagnosis rate was defined as the number of people newly diagnosed with HIV for every 100,000 people attended.

For descriptive analyses, we used absolute and relative frequencies, and means and standard deviations, or medians with an interquartile range (IQR). Comparison of categorical variables was performed using the chi-squared test (or Fisher's exact test if necessary), and the Student’s t-test was used for means comparison. Binary logistic multivariable models were created to evaluate the effect of independent variables (gender, origin, age, and location) on the results of the RE&IC questionnaire and subsequent risk of HIV diagnosis. All contrasts were bilateral and assumed confidence intervals (CI) of 95%. P values < 0.05 were considered to be statistically significant. Statistical analysis was performed using Stata 15.1 software (Stata Corp-LP, College Station, TX), and all analyses were carried out in collaboration with the Biostatistics Department of the Ramon y Cajal Hospital.

## Results

### DRIVE program: Baseline characteristics, testing coverage, and new HIV diagnoses

A total of 5,329 participants were included in the DRIVE program, of whom 2,684 (50.4%) were women. The median age was 37 (IQR: 28–47) years old. Spaniards made up 74.9% of participants, and 69.3% of HIV tests were performed in the primary care center. Only 4 participants were excluded: 2 came out as false positives, while 2 indeterminate. No cases were excluded because of invalid questionnaires. Three other participants were excluded because a prior diagnosis was identified. Table 1 shows the baseline characteristics of participants and the comparison between the emergency department and the primary care center.

**Table 1:** Participant characteristics according to the healthcare setting in the DRIVE program.

	Primary care center (n = 3696)	Emergency Department (n = 1631)	OR (95% CI)	*p*
**Male gender, %**	51.5	45.6	1.27 (1.13–1.42)	< 0.001
**Age, %**				
**< 30 years**	27.4	28.3	-	-
**30–50 years**	52.7	52.6	1.03 (0.90–1.18)	0.634
**> 50 years**	19.8	19.1	1.07 (0.90–1.27)	0.424
**Origin, %**				
**Spain**	74.1	76.9	-	-
**Latin America**	22.0	15.8	1.45 (1.24–1.69)	< 0.001
**Africa**	0.7	1.7	0.44 (0.26–0.76)	0.003
**Eastern Europe**	2.1	3.4	0.65 (0.46–0.92)	0.015
**Western-Central Europe**	0.6	1.5	0.44 (0.25–0.78)	0.004
**Other**	0.4	0.7	0.67 (0.31–1.44)	0.306
Previous visit to primary care center*, %	96.7	89.1	3.57 (2.81–4.52)	< 0.001
**Previous HIV testing, %**	32.5	23.7	1.54 (1.35–1.76)	< 0.001
**Positive RE&IC questionnaire, %**	49.4	55.4	0.78 (0.70–0.88)	< 0.001
**Positive HIV test, n (rate per 100 tests performed)**	8 (0.22)	14 (0.86)	0.25 (0.11–0.60)	0.001

CI: confidence interval; OR: odds ratio; RE&IC: risk of exposure and indicator conditions

*Visit within two years prior to inclusion in the study.

All participants filled in the RE&IC questionnaire, and more than half (51.2%) reported at least one positive answer. Both men and younger individuals were more likely to present a positive result, as were those who had previous contact with primary care, patients already tested for HIV in the past, and participants included from the emergency department (Table 2).

**Table 2:** Results of the RE&IC questionnaire in the DRIVE program, according to respondent characteristics.

	Positive questionnaire* (n = 2731)	*p*	Multivariable analysis	
			Adjusted OR^¶^ (95% CI)	*p*
**Gender, %**				
**Male (n = 2648)**	54.3	< 0.001	1.37 (1.23–1.53)	< 0.001
**Female (n = 2685)**	48.1			
**Age, %**				
**< 30 years (n = 1477)**	55.0	-	-	-
**30–50 years (n = 2810)**	51.0	0.012	0.76 (0.68–0.88)	< 0.001
**> 50 years (n = 1045)**	46.5	< 0.001	0.70 (0.59–0.82)	< 0.001
**Origin, %**				
**Spain (n = 3995)**	51.4	-	-	-
**Latin America (n = 1073)**	49.8	0.345		
**Africa (n = 53)**	58.5	0.306		
**Eastern Europe (n = 135)**	52.6	0.783		
**Western-Central Europe (n = 49)**	59.2	0.280		
**Other (n = 27)**	44.4	0.473		
**Previous visit to primary care center^§^, %**				
**Yes (n = 5031)**	51.6	0.027	1.40 (1.10–1.78)	0.006
**No (n = 302)**	45.0			
**Previous HIV testing, %**				
**Yes (n = 1588)**	60.6	< 0.001	1.85 (1.64–2.09)	< 0.001
**No (n = 3741)**	47.2			
**Study setting, %**				
**Primary care center (n = 3696)**	49.4	< 0.001	0.71 (0.63–0.80)	< 0.001
**Emergency department (n = 1637)**	55.4			

CI: confidence interval; OR: odds ratio; RE&IC: risk of exposure and indicator conditions

* A questionnaire was considered positive if any answered item was ‘yes.’

^**¶**^ Adjusted by gender, age, previous visits to primary care, previous HIV testing, and setting of inclusion in the study.

^**§**^ Visit within two years prior to inclusion in the study.

Twenty-two HIV-positive people were identified, which corresponds to an overall new HIV diagnosis rate of 4.1‰, with a higher percentage in the emergency department than the primary care center (8.6‰ vs. 2.2‰; p = 0.001). All of them had at least one positive answer on the RE&IC questionnaire. The new HIV diagnosis rate was higher among men (6.8‰ vs. 1.5‰; p = 0.002), and foreigners (7.5‰ vs. 3‰; p = 0.027). Individuals from Eastern Europe and previously HIV-tested participants were more likely to be diagnosed with HIV, as summarized in Table 3.

**Table 3:** Differences between participants according to HIV test results.

	HIV-positive (n = 22)	OR (95% CI)	*p*
**Gender, n (%)**			
**Male (n = 2648)**	18 (0.7)	4.59 (1.55–13.6)	0.002
**Female (n = 2688)**	4 (0.1)		
**Age, n (%)**			
**< 30 years (n = 1477)**	6 (0.4)	-	-
**30–50 years (n = 2810)**	12 (0.4)	1.05 (0.39–2.81)	0.919
**> 50 years (n = 1045)**	4 (0.4)	0.94 (0.27–3.35)	0.929
**Origin, n (%)**			
**Spain (n = 3995)**	12 (0.3)	-	-
**Latin America (n = 1073)**	7 (0.7)	2.18 (0.86–5.56)	0.102
**Africa (n = 53)**	0 (0)	-	0.998
**Eastern Europe (n = 135)**	3 (2.2)	7.54 (2.10–27.0)	0.002
**Western-Central Europe (n = 49)**	0 (0)	-	0.998
**Other (n = 27)**	0 (0)	-	0.998
**Previous visit to primary care center^§^, n (%)**			
**Yes (n = 5027)**	20 (0.4)	0.60 (0.14–2.58)	0.357
**No (n = 302)**	2 (0.7)		
**Previous HIV testing, n (%)**			
**Yes (n = 1586)**	13 (0.8)	3.42 (1.46–8.03)	0.004
**No (n = 3739)**	9 (0.2)		

CI: confidence interval; OR: odds ratio

^**§**^ Visit within the two years prior to inclusion in the study.

### Routine clinical practice vs. the DRIVE program: Testing coverage and new HIV diagnoses

The estimated HIV testing coverage was significantly higher in the DRIVE program than in routine clinical practice (7.17% vs. 0.96%, p < 0.001); it was better in the primary care center than in the emergency department, given the two strategies. The rate of new HIV diagnoses was also better in the DRIVE program (29.6 vs. 3.1 per 100,000 patients attended, p < 0.001), as shown in Table 4.

**Table 4:** Estimated HIV testing coverage and new HIV diagnoses rates, according to the observation period and the healthcare setting.

	DRIVE program 2012–2013	Clinical Practice 2011–2014	OR (95% CI)	*p*
**Emergency Department *(attended population in one year = 63*,*054)***				
**Tested population, n**	1635	966	-	-
**Testing coverage, %**	2.59	0.51	5.1 (4.7–5.5)	< 0.001
**New HIV diagnoses, n (rate per 100,000)**	14 (22.2)	6 (3.2)	7.0 (2.7–18.2)	< 0.001
**Primary care center *(attended population in one year = 11*,*220)***				
**Tested population, n**	3694	1185	-	-
**Testing coverage, %**	32.92	3.52	9.4 (8.8–9.9)	< 0.001
**New HIV diagnoses, n (rate per 100,000)**	8 (71.3)	1 (3.0)	24.0 (3.0–191.9)	< 0.001
**Overall *(attended population in one year = 74*,*274)***				
**Tested population, n**	5329	2152	-	-
**Testing coverage, %**	7.17	0.97	7.4 (7.1–7.8)	< 0.001
**New HIV diagnoses, n (rate per 100,000)**	22 (29.6)	7 (3.1)	9.4 (4.0–22.1)	< 0.001

CI: confidence interval; OR: odds ratio

### Immunological status at diagnosis

Participants within the DRIVE program showed a non-significant higher median baseline CD4 count than those diagnosed in routine clinical practice (263 vs. 177 cells/mm^3^, respectively) (p = 0.901). Late HIV diagnosis, defined as the presence of a baseline CD4 cell count ≤ 350 cells/mm^3^ or the presence of an AIDS-defining condition at diagnosis, was higher in routine clinical practice despite being non-statistically significant (54.5% vs. 71.4%, p = 0.665); this included advanced HIV disease (≤ 200 cells/mm^3^), with 40.9% in the DRIVE program vs. 57.1% in routine clinical practice (p = 0.667). The median CD4 cell count at diagnosis was higher among patients diagnosed in the primary care center than those diagnosed in the emergency department (481 vs. 194 cells/mm^3^, p = 0.114). Patients diagnosed in the emergency department presented a trend towards a higher late HIV diagnosis rate (70% vs. 33%, p = 0.106), exhibiting smaller differences in the percentage of advanced disease at diagnosis (50% vs. 33.3%, p = 0.454).

According to risk groups, men who have sex with men (MSM) were diagnosed earlier (median CD4 cell count 391 cells/mm^3^) than heterosexual men and women (207 cell/mm^3^) or injected drug users (82 cell/mm^3^) (p = 0.060). Thus, late diagnosis was lower among MSM than the other two groups combined (38.5% vs. 77.8%, p = 0.099). There were no differences in the percentage of late diagnosis according to the level of education (p = 0.974) or gender (p = 0.370).

## Discussion

The DRIVE study is one of the largest HIV testing programs in Spain over the last several years. It highlights the benefits of performing structured HIV screening, in terms of improved testing coverage, number of HIV diagnoses, and late diagnosis rates.

Testing for HIV is currently one of the main components of the strategies to control the HIV epidemic. The European region of the World Health Organization (WHO) is close to achieving the UNAIDS 90-90-90 target [31], with the main difficulty in terms of reaching the first 90 [32]. Therefore, the development of HIV testing programs is essential to meet the proposed objectives. During the first years of the epidemic, HIV-risk screening instruments [33] and self-reported sexual behavior risk factors [34] were evaluated, mainly for public health purposes. In recent years, HIV infection risk assessment has been more extensively studied. A score based on epidemiological characteristics and risk behaviors was shown to accurately categorize patients into groups with increasing probabilities of HIV infection [35], and was demonstrated to be strongly associated with new HIV diagnoses compared to non-targeted screening in the emergency department [23]. In line with the European Centre for Disease Prevention and Control (ECDC) guidance [36], some studies show that indicator conditions guiding HIV testing is an effective strategy to address the HIV epidemic in Europe as well as reducing the number of undiagnosed HIV infections [29,30]. Our questionnaire is the first tool to date that assesses the risk of HIV infection by combining the risk of exposure and indicator conditions, thus improving its accuracy [28]. According to UNAIDS estimations, 1.5 to 2.3 million people are living with HIV in Latin America [37], most of them Spanish-speaking. Spanish is used by 370 million people across the globe, but until our study, there was not a validated questionnaire in Spanish to assess the risk of HIV infection [28].

The DRIVE program, through the use of the RE&IC questionnaire, has achieved a testing coverage which is more than five times higher than non-targeted screening. Men, people aged 30–50 years, and participants with previous contact with primary care or who were previously tested for HIV were more likely to present a positive risk questionnaire. This is consistent with the population groups in which the epidemic is currently concentrated in our setting [5], and indicates that this tool could easily discriminate the patients who are most at risk according to the current guidelines [36]. Testing coverage in the DRIVE program was much higher in the primary care center than the emergency department. Primary care centers are most frequently used by the Spanish population to carry out HIV screening [26], and numerous studies agree about the high prevalence of missed opportunities for HIV testing in this setting [12,38]; implementation of HIV testing programs, such as DRIVE, would have a tremendous impact on screening rates and the reduction of missed diagnostic opportunities. A potentially useful strategy to increase coverage in emergency room scenarios might be the automatic implementation of the questionnaire within the electronic medical screening system, as it has been done in previous studies showing good results [23].

The use of rapid tests and nurse participation has facilitated the development of the screening; as such, immediate results are obtained, counseling is provided, and patients are referred to the HIV Infectious Diseases Service in a single visit. The implementation of HIV early diagnosis programs through the use of rapid tests in primary care centers has been a pioneering intervention in Spain [39]. These strategies promote the screening of high percentages of the population at risk. Previous studies demonstrated a similar diagnostic capacity for rapid tests (if performed correctly) and conventional diagnosis by ELISA and Western Blot. Virtually all blood rapid diagnostic tests reach a sensitivity and specificity close to 100%, although erroneous results may still appear [40].

The DRIVE program achieved a rate of new diagnoses seven times higher than in routine clinical practice, with a percentage of new HIV diagnoses higher than expected, according to the prevalence of undiagnosed HIV infection reported in previous studies in our healthcare area [41]. New HIV diagnoses were more frequent among men, Eastern Europeans (in line with the current epidemiological data [1]), and people previously tested for HIV. The latter may indicate a greater self-awareness of the risk of HIV infection, or an increased detection of risk in this population by healthcare providers. The rate of new HIV diagnoses out of 1000 tests performed was higher in the emergency department, which may be related to the greater risk of HIV exposure and indicator conditions reported by participants in this setting. This data corroborates that emergency departments should not be excluded from HIV screening programs, as indicated in the CDC testing guidelines [36].

The higher HIV infection prevalence in certain population groups reflects an ongoing stereotype about at-risk profiles; however, we should not neglect the risk that also exists in other groups, such as women and the elderly, where higher percentages of late diagnosis are observed [42]. In our study, heterosexual men and women were diagnosed with a lower CD4 cell count than MSM, although the small sample size has not made it possible to obtain statistically significant differences. Late diagnosis was lower in our structured testing program than in routine clinical practice, indicating that broad screening could improve current rates of late diagnosis, which reach almost 50% in Europe [5]. Regarding the site of inclusion in the study, the emergency department represents the most common facility for late diagnosis in our cohort, which has been shown in other studies that place the emergency department as the site where diagnostic opportunities are most frequently missed [43]. As such, both the emergency departments and primary care centers are healthcare settings where more effort should be made to improve HIV screening, as indicated by international testing guidelines [36].

Our study has some limitations. It is a prospective observational single-center study, with retrospective control arm, in which external resources were used to screen for HIV infection. This could decrease its external validity, so further research is necessary to assess the applicability of the screening program in daily life.

In conclusion, the development of a simple screening program, through the use of a RE&IC questionnaire an rapid HIV tests, could achieve an increase in the rates of HIV testing, an increase in new HIV diagnoses, as well as an improvement in the current rates of late diagnosis. This strategy might be a useful tool for decreasing undiagnosed HIV infection and ultimately mitigating the epidemic.
